# Idiopathic Multicentric Castleman Disease Associated With Seronegative Erosive Chronic Polyarthritis: A Diagnostic Challenge

**DOI:** 10.1002/ccr3.73520

**Published:** 2026-09-15

**Authors:** Birahim Lo, Baïdy Sy Kane, Fatine Benjelloun, Mouhamed Almakhy Niang, Ibra Kaïré, Charifah Siddiki, Avotra Razafimandimby, Hamidou Deme, Cherif Mouhamed Moustapha Dial, Abdoulaye Pouye

**Affiliations:** ^1^ Department of Internal Medicine Cheikh Anta Diop University Dakar Senegal; ^2^ Department of Radiology Cheikh Anta Diop University Dakar Senegal; ^3^ Department of Pathology Cheikh Anta Diop University Dakar Senegal

**Keywords:** Castleman disease, idiopathic multicentric Castleman disease, immunohistochemistry, lymphadenopathy, seronegative polyarthritis, synovitis

## Abstract

Idiopathic multicentric Castleman disease can rarely present with systemic inflammation and erosive seronegative polyarthritis. Awareness of this association helps prevent misdiagnosis and ensures appropriate immunosuppressive management based on clinicopathological and immunohistochemical correlation. Careful multidisciplinary evaluation is essential to distinguish this uncommon presentation from autoimmune and lymphoproliferative disorders in clinical practice.

## 1 Introduction

Castleman disease (CD) is a benign lymph node pathology characterized by particular, though non‐specific, histology, notably angiofollicular hyperplasia, associated with clinical and biological elements [1, 2]. Since the first descriptions by Benjamin Castleman in the 1950s of what became unicentric CD, the clinical and pathological spectrum has evolved considerably [3]. Currently, three major clinicopathological forms of CD are described as distinct entities sharing common lymph node histological changes: unicentric CD, often asymptomatic, mainly affecting children and young adults; multicentric CD associated with human herpesvirus 8 (HHV‐8), linked in 50% of cases to HIV coinfection; and idiopathic multicentric Castleman disease (iMCD) involving multiple lymph node regions and possibly spleen, bone marrow, and extranodal sites [4].

Although CD is primarily characterized by lymph node and systemic manifestations, extranodal involvement may occur. Joint involvement is rare and has scarcely been reported in the literature. One study described a case of polyarthritis with active rheumatoid‐like synovitis in a patient with iMCD treated with prednisone [5].

Herein, we report a case of iMCD associated with seronegative and erosive chronic polyarthritis.

## 2 Case Presentation/Examination

**FIGURE 1:**
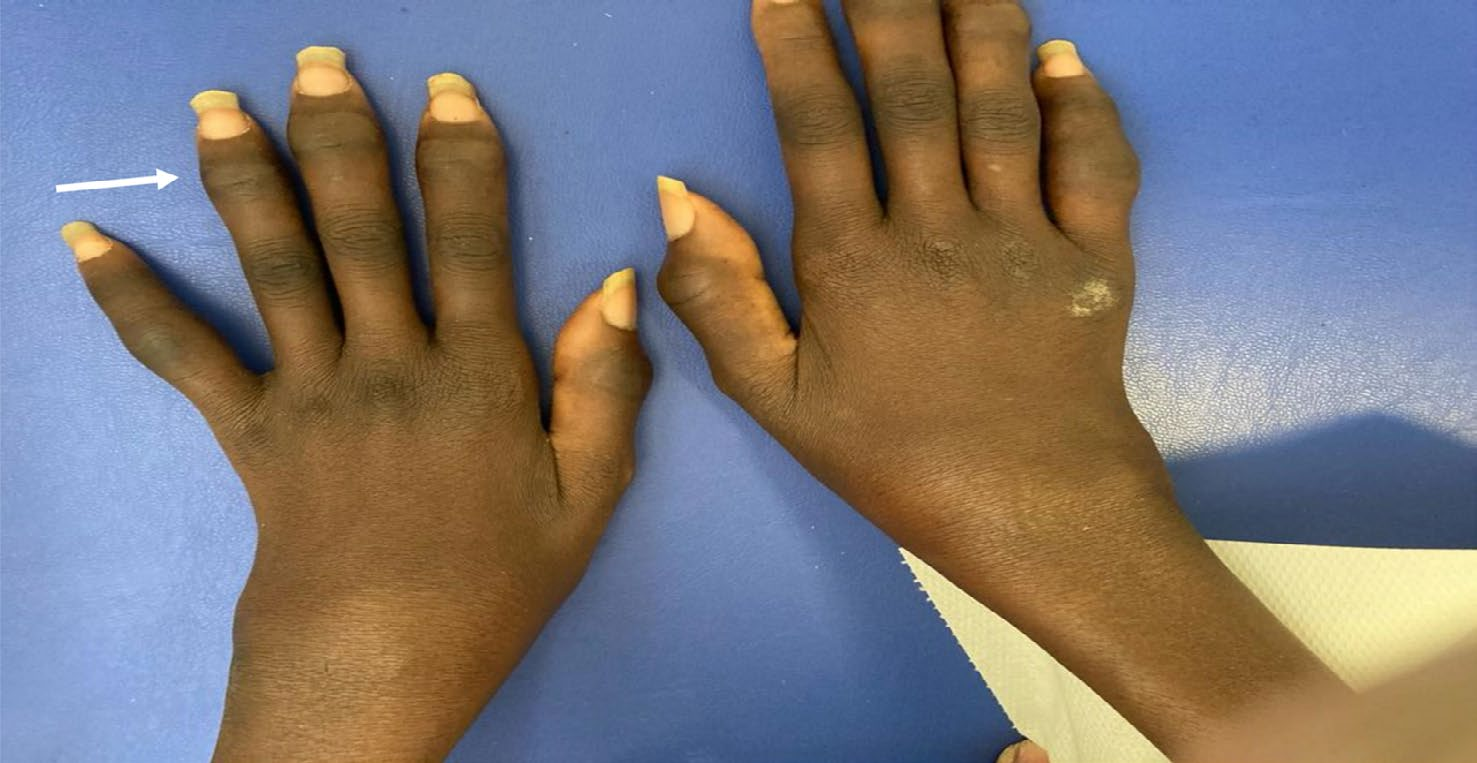
Polyarthritis with “carrot‐like” finger appearance.

A 23‐year‐old woman with no significant past medical history was referred from the emergency department for evaluation of chronic polyarthralgia. On admission, she presented with non‐deforming peripheral polyarthritis involving both small and large joints (Figure 1), without psoriasis or clinical enthesopathy. She also exhibited bilateral asymmetric cervical, axillary, and inguinal lymphadenopathy, the largest measuring 3 cm in the left axilla, a fever of 38.6°C, and well‐tolerated anemic syndrome without externalized bleeding. Her vital signs on admission were: blood pressure 109/78 mmHg, heart rate 118 bpm, respiratory rate 25 breaths/min, oxygen saturation 97%. Her weight was 30 kg in the context of unquantified disease‐related weight loss. Her height and baseline BMI were not available.

Initial laboratory investigations showed a nonspecific inflammatory syndrome with C‐reactive protein of 47.50 mg/L, an erythrocyte sedimentation rate of 130 mm (first hour), hypochromic microcytic anemia of 7.4 g/dL and a serum ferritin level of 4698.20 μg/L.

## 3 Methods

**FIGURE 2:**
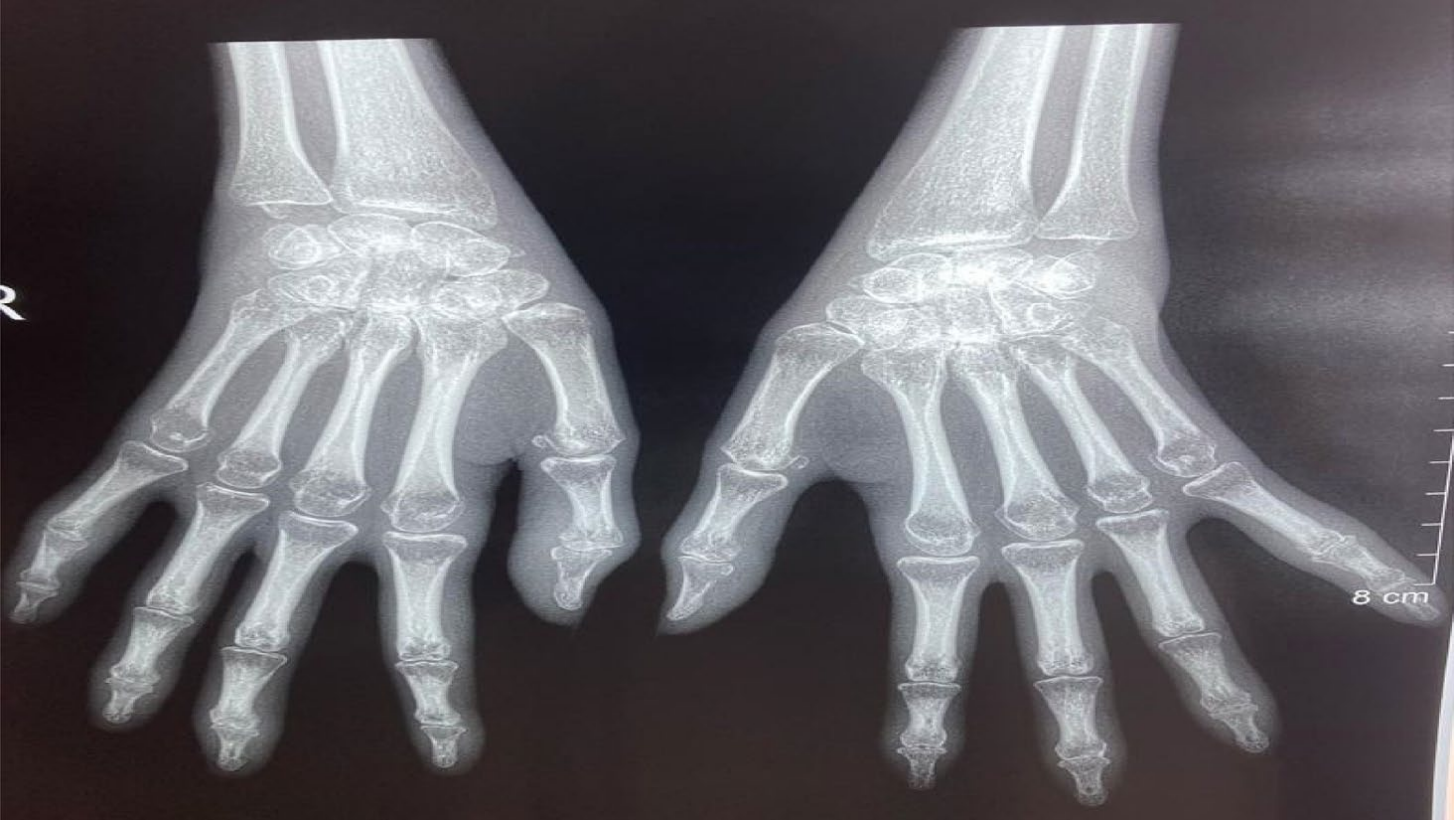
Hand radiograph showing diffuse demineralization and soft tissue thickening around the distal interphalangeal joints.

**FIGURE 3:**
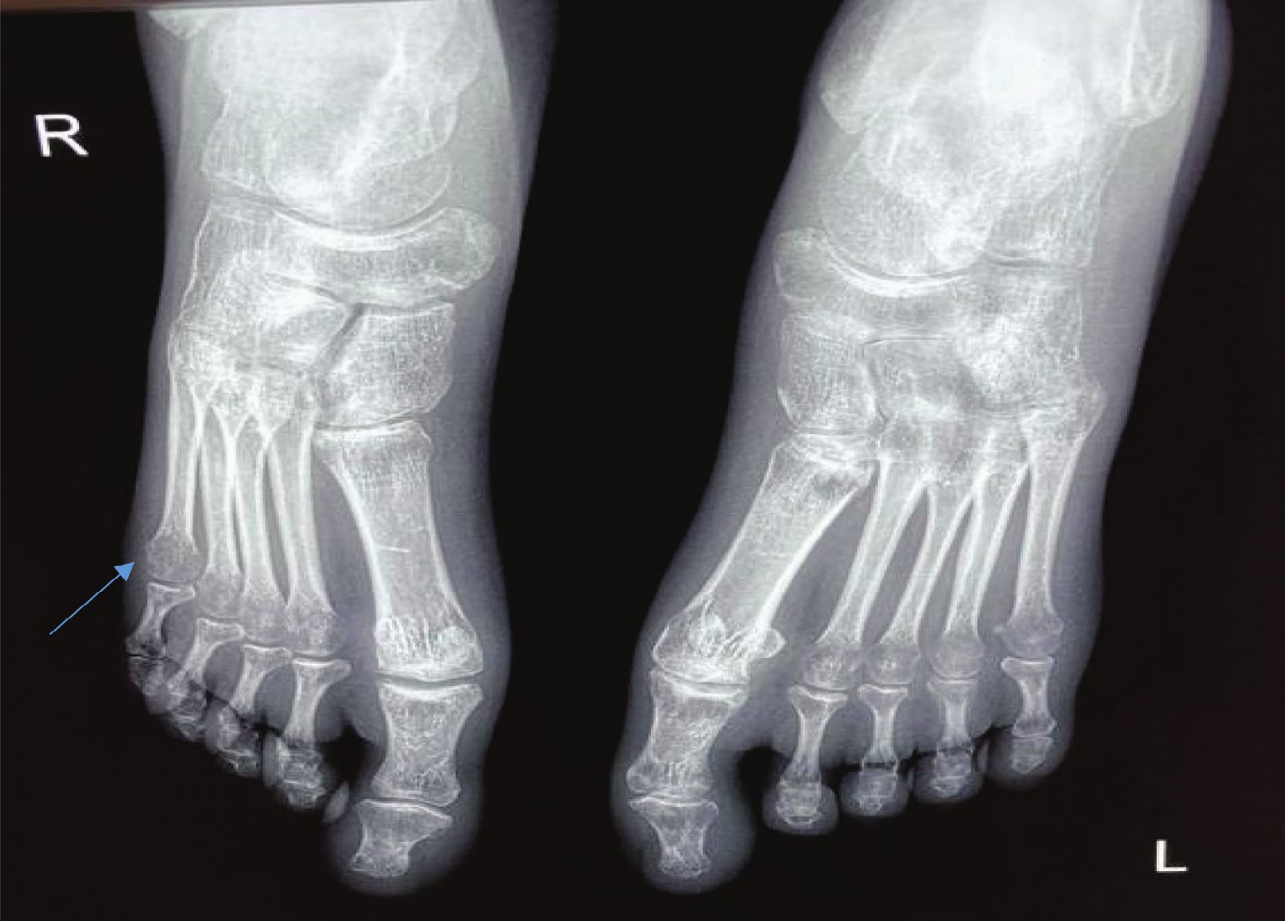
Diffuse bone demineralization with erosion of the 5th metatarsophalangeal head (arrow).

**FIGURE 4:**
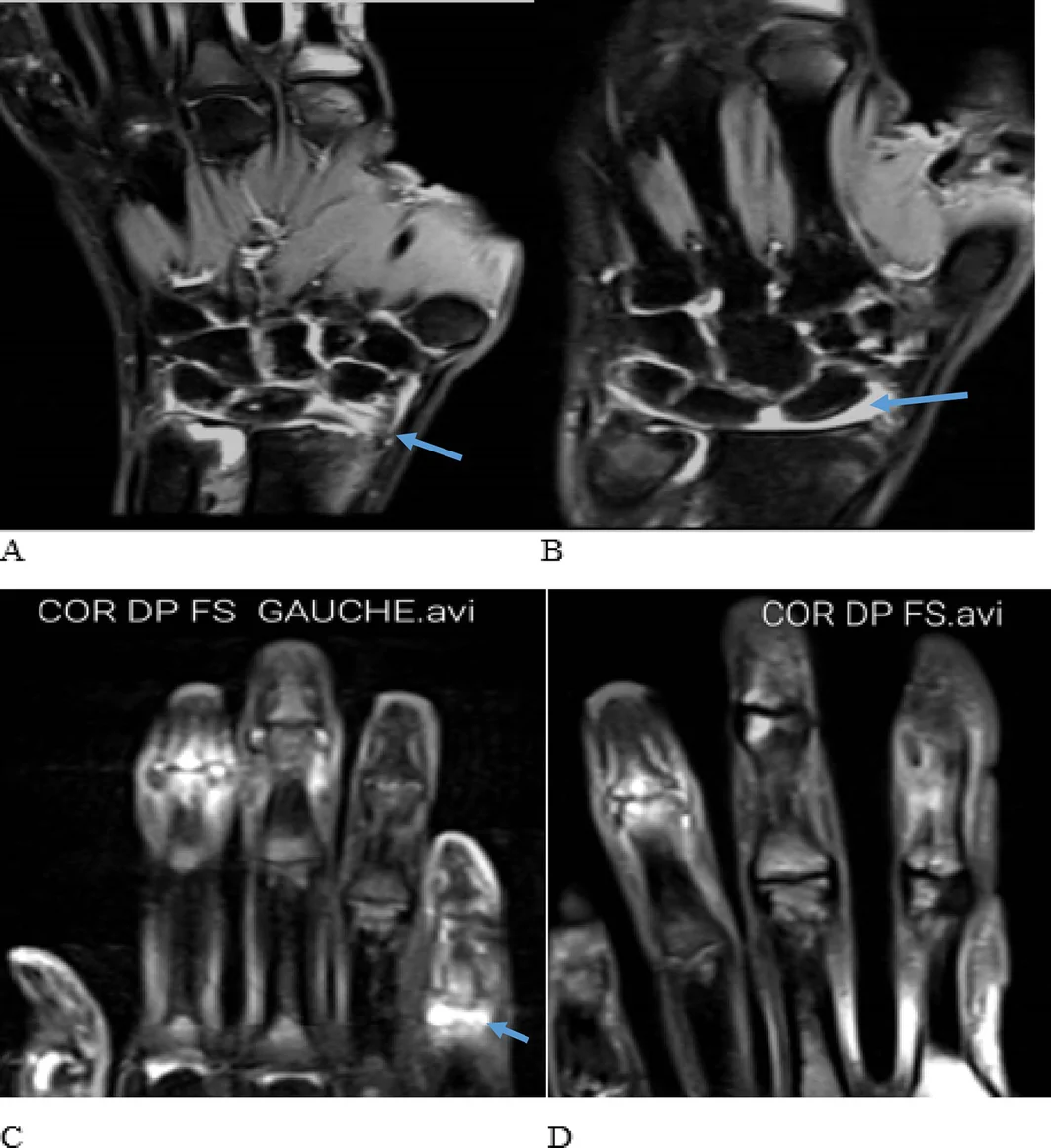
MRI of the hands and wrists using DP FAT‐SAT sequences showing bone erosions of the distal end of the radius (A, arrow), intra‐articular radiocarpal effusion (B, arrow), and erosions with intra‐articular effusion of the DIP joints (C, arrow; D), consistent with synovitis.

Given the clinical picture of chronic polyarthritis, anti‐citrullinated protein antibodies and antinuclear antibodies were requested and found negative. Radiographs revealed diffuse demineralization (Figure 2) and erosions (Figure 3), and ultrasound showed erosions. MRI of her hands and wrists showed synovitis, erosions, and hypersignal edema localized in certain interphalangeal joints (Figure 4). A diagnosis of seronegative erosive chronic polyarthritis was established.

**FIGURE 5:**
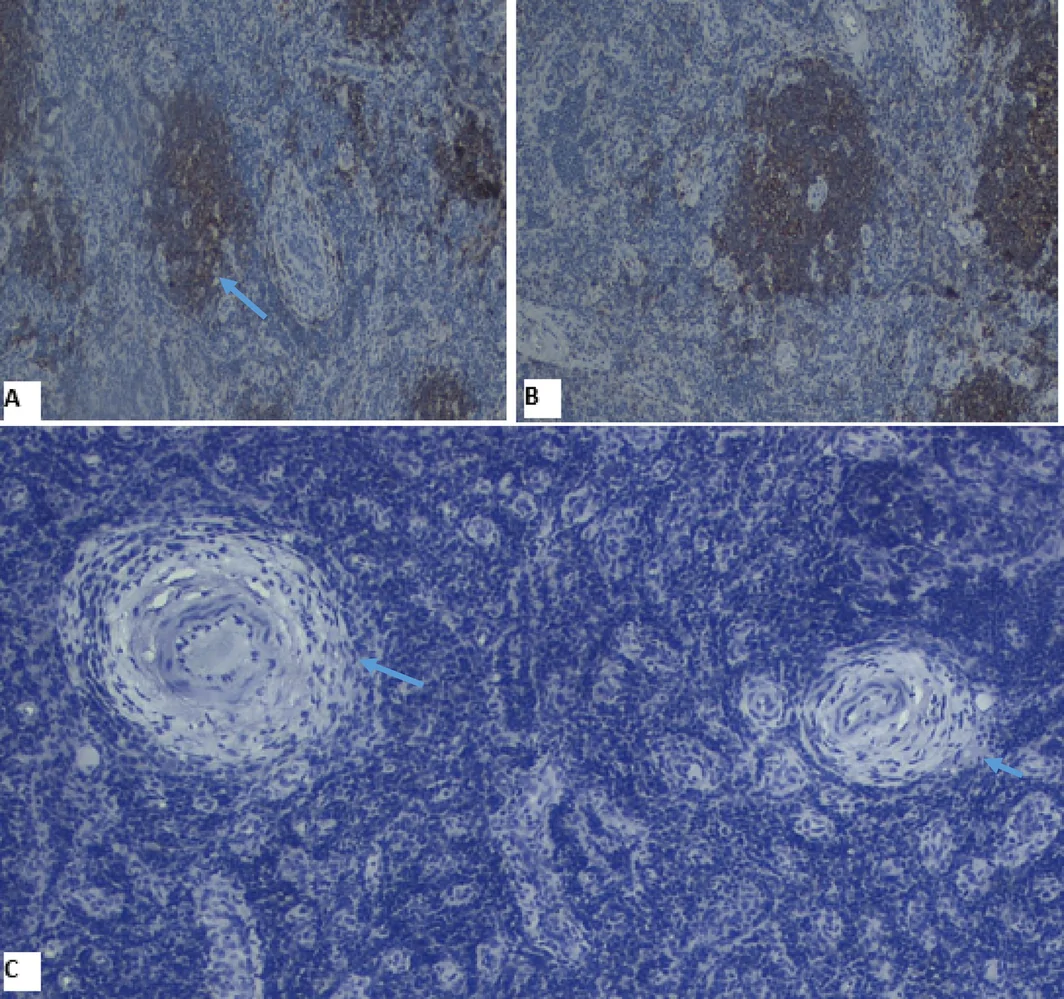
Histology of the patient's Castleman disease. (A, B) CD20 positive follicles with abundant vessels and hyalinized walls. The arrow indicates a vessel with a hyalinized wall. (C) Two vessels with hyalinized walls (arrows). Immunohistochemistry showing germinal center positivity for CD3, and Ki67. Strong plasmacytic positivity highlighted by CD138 staining. LM91 and HHV8 stainings are negative. Magnification ×100.

Due to lymphadenopathy, a lymph node biopsy was performed. The biopsy revealed a node homogenized by diffuse lymphoid proliferation of large cells. These cells had basophilic cytoplasm and large nuclei with a single prominent central nucleolus or three to five nucleoli adjacent to the nuclear membrane. These corresponded to immunoblasts and centroblasts respectively. The cells were arranged in diffuse sheets with scant interstitial tissue. The subcapsular sinus was infiltrated; the capsule was intact and slightly thickened. Initial histological diagnosis suggested diffuse large B‐cell lymphoma but immunohistochemistry concluded reactive follicular and diffuse lymphoid hyperplasia. A biopsy re‐evaluation was requested with additional immunohistochemical studies to search for atypical lymphoproliferative syndrome. This review showed diffuse follicular lymphoid hyperplasia associated with capillaro‐venous vascular proliferation and parietal hyalinization. These vessels occupied the interfollicular areas and extended into the germinal centers, supporting the diagnosis of CD. Immunohistochemistry showed negative HHV‐8 staining and numerous CD138‐positive plasma cells (Figure 5).

The underlying immunodepression was investigated. Retroviral serology was negative and the CD4 count was 835 cells/μL with a CD4/CD8 ratio of 1.30. Serum protein electrophoresis showed polyclonal hypergammaglobulinemia. These findings classified the disease as iMCD.

Additional workup included negative GeneXpert on gastric lavage, negative hepatitis B surface antigen, Lactate dehydrogenase of 826 UI/L, and sterile blood and urine cultures.

The patient was treated with oral corticosteroids. Because of persistent, although markedly improved, DIP synovitis, methotrexate (10 mg/week) was introduced at the fifth month of follow‐up as a steroid‐sparing agent, allowing gradual tapering of prednisone to 5 mg every other day.

## 4 Conclusion and Results

Clinical evolution was favorable, with regression of peripheral lymphadenopathy and polyarthralgia. Baseline laboratory findings and their evolution during follow‐up are summarized in Table 1.

**TABLE 1:** Baseline and follow‐up laboratory findings.

Laboratory parameter	At diagnosis	Last follow‐up
Hemoglobin (g/dL)	7.4	13.7
White blood cell count (×10^9^/L)	10.1	4.6
Erythrocyte sedimentation rate (1st hour, mm)	130	NR
C‐reactive protein (mg/L)	47.5	6
Ferritin (μg/L)	4698.2	NR
Gamma globulins (g/L)	29.9	15
Serum protein electrophoresis	Polyclonal hypergammaglobulinaemia	Improved
Antinuclear antibodies (ANA)	Negative	NR
Rheumatoid factor	Negative	NR
Anti‐cyclic citrullinated peptide (anti‐CCP) antibodies	Negative	NR
Extractable nuclear antigen (ENA) antibodies	Negative	NR
Anti‐double‐stranded DNA (anti‐dsDNA) antibodies	Negative	NR

Abbreviation: NR, not repeated.

Persistent synovitis of the distal interphalangeal (DIP) joints was observed despite resolution of fever and normalization of hemoglobin. At the last follow‐up, 24 months after diagnosis, no recurrence of peripheral lymphadenopathy was observed, and the patient remained clinically stable with mild persistent active arthritis limited to residual DIP synovitis. Her weight had increased from 30 kg at diagnosis to 42 kg at the last clinical assessment.

## 5 Discussion

The diagnosis of HHV‐8‐negative/iMCD requires two major criteria (multicentric lymphadenopathy and lymph node histopathology consistent with the iMCD spectrum), at least two of the 11 minor criteria, including at least one laboratory abnormality, and exclusion of infectious, malignant, and autoimmune disorders that could better explain the clinical presentation [6]. Our patient fulfilled these diagnostic criteria and was classified as having iMCD‐NOS, as she did not exhibit the defining clinical features of TAFRO syndrome, including true thrombocytopenia, anasarca, renal dysfunction, or reticulin myelofibrosis [6, 7].

The case reported here concerns a 23‐year‐old woman with no notable history presenting iMCD associated with seronegative erosive chronic polyarthritis. This rare association highlights the importance of considering atypical systemic disease forms when clinical and biological symptoms do not perfectly fit classic diagnostic criteria.

CD is a benign lymphoproliferative pathology characterized by diffuse lymphoid hyperplasia, often associated with inflammatory symptoms including lymphadenopathy, fever, and elevated inflammatory markers. The markedly elevated ferritin level (4698.2 μg/L) in our patient was considered a nonspecific marker of the pronounced systemic inflammatory response. It is classified mainly into unicentric and multicentric forms. The idiopathic multicentric form, as observed in our patient, is the least frequent and typically occurs in immunocompetent individuals without evident underlying immune dysfunction. This case highlights the diagnostic complexity of iMCD, where the absence of an infectious cause or immunodeficiency complicates the initial diagnosis [8].

The absence of HHV‐8 staining, together with the characteristic histopathological findings and the absence of immunodeficiency, supported the diagnosis of iMCD. Although numerous CD138‐positive plasma cells were present, the overall clinicopathological features were consistent with iMCD. The histopathological features observed, notably capillaro‐venous vascular proliferation and parietal hyalinization, are typical of this form and help distinguish it from lymphomas or other lymphoproliferative disorders [9].

Although exclusion of autoimmune diseases is required when establishing the diagnosis of iMCD, this criterion aims to exclude patients in whom an autoimmune disease better explains the clinical presentation. Rarely, inflammatory arthritis may coexist with iMCD, as observed in our patient after careful exclusion of connective tissue diseases and rheumatoid arthritis.

The association of CD with chronic inflammatory polyarthritis is extremely rare and has been reported in isolated cases [5]. Our patient developed a peripheral, polysynovial polyarthritis mainly involving small joints, particularly the distal interphalangeal joints, without psoriasis or enthesopathy. Although polyarthritis in CD is often nonspecific, the present clinical presentation suggested erosive inflammatory polyarthritis associated with a systemic inflammatory syndrome.

The clinical picture of erosive polyarthritis coupled with lymphadenopathy initially suggested seronegative erosive chronic polyarthritis associated with a lymphoproliferative syndrome. The diagnosis of rheumatoid arthritis was considered unlikely due to clinical presentation with predominance of DIP synovitis. Furthermore, autoimmune screening, including rheumatoid factor, anti‐CCP antibodies, ANA, ENA antibodies, and anti‐dsDNA antibodies, was negative. Musculoskeletal ultrasonography, radiography, and MRI confirmed erosive inflammatory arthritis in our patient, as previously reported in rare cases of iMCD‐associated arthritis [5].

This association suggests that systemic inflammation linked to CD could induce inflammatory joint symptoms and erosive lesions, although the mechanism remains poorly understood. It is essential to consider that CD may sometimes cause musculoskeletal manifestations due to increased production of inflammatory cytokines and immune axis stimulation [9].

Treatment of iMCD currently relies on anti‐interleukin‐6 (IL‐6) therapy as the recommended first‐line treatment for most patients with iMCD [7, 10]. However, access to IL‐6 inhibitors remains limited in many low‐resource settings. In our patient, oral corticosteroids induced rapid regression of peripheral lymphadenopathy and improvement of polyarthritis. Because of persistent, although markedly improved, synovitis of the distal interphalangeal joints, methotrexate (10 mg/week) was subsequently introduced as a steroid‐sparing agent, allowing gradual tapering of prednisone to 5 mg every other day. This therapeutic strategy resulted in sustained clinical improvement without recurrence of peripheral lymphadenopathy during follow‐up.

The persistence of synovitis in the DIP joints despite improvement in other clinical and biological parameters suggested a more refractory articular involvement. Additional therapeutic options may therefore be required, including conventional immunosuppressive drugs or targeted therapies. Recent evidence has also identified TNF inhibition as a potential therapeutic strategy, particularly in patients with the TAFRO subtype of iMCD [11].

This case underlines the need for thorough differential diagnosis when erosive polyarthritis accompanies lymphadenopathy and emphasizes not excluding rare systemic diseases such as CD, especially in the absence of classical criteria. It is important to note the limitations of a single case report. Although the clinical evolution was favorable under corticosteroid therapy, this report remains limited by its single‐case design. Multicentric studies are needed to better understand the relationship between CD and musculoskeletal disorders and to determine optimal therapeutic strategies in these atypical cases.

Finally, this case stresses the importance of close collaboration between clinicians and pathologists in diagnosing complex diseases. Initially, the lymph node biopsy pointed to diffuse large B‐cell lymphoma. However, it was through detailed review and complementary immunohistochemical study that the diagnosis was adjusted to iMCD. This re‐evaluation highlights how essential it is to discuss pathological findings collaboratively to refine diagnoses, especially when similar diseases may cause misdiagnosis. Team review also helps avoid such misdiagnoses and inappropriate treatment, particularly when clinical manifestations are atypical and overlap with other lymphoproliferative disorders. Continuous communication between clinicians and pathologists remains a key factor for accurate diagnosis and optimal patient care.

## Author Contributions

**Birahim Lo:** conceptualization, data curation, formal analysis, investigation, methodology, project administration, resources, software, supervision, validation, visualization, writing – original draft, writing – review and editing. **Baïdy Sy Kane:** conceptualization, methodology, project administration, supervision, validation, visualization, writing – review and editing. **Fatine Benjelloun:** conceptualization, methodology, resources, validation, writing – original draft, writing – review and editing. **Mouhamed Almakhy Niang:** conceptualization, writing – review and editing. **Ibra Kaïré:** software, validation, visualization, writing – review and editing. **Charifah Siddiki:** conceptualization, validation, visualization. **Avotra Razafimandimby:** resources, software, writing – review and editing. **Hamidou Deme:** resources, software, validation, visualization. **Cherif Mouhamed Moustapha Dial:** data curation, resources, validation, visualization, writing – review and editing. **Abdoulaye Pouye:** supervision, validation, writing – review and editing.

## Funding

The authors have nothing to report.

## Consent

Written informed consent was obtained from the patient for publication of this case report and accompanying images.

## Data Availability

The data supporting the findings of this case report are available within the article. Additional de‐identified clinical data are available from the corresponding author upon reasonable request, provided this is consistent with patient confidentiality and institutional policies.
